# The Potential of Chatbots for Emotional Support and Promoting Mental Well-Being in Different Cultures: Mixed Methods Study

**DOI:** 10.2196/51712

**Published:** 2023-10-20

**Authors:** Hyojin Chin, Hyeonho Song, Gumhee Baek, Mingi Shin, Chani Jung, Meeyoung Cha, Junghoi Choi, Chiyoung Cha

**Affiliations:** 1 Data Science Group Institute for Basic Science Daejeon Republic of Korea; 2 School of Computing Korea Advanced Institute of Science and Technology Daejeon Republic of Korea; 3 College of Nursing and Ewha Research Institute of Nursing Science System Health & Engineering Major in Graduate School Ewha Womans University Seoul Republic of Korea; 4 SimSimi Inc Seoul Republic of Korea

**Keywords:** chatbot, depressive mood, sad, depressive discourse, sentiment analysis, conversational agent, mental health, health information, cultural differences

## Abstract

**Background:**

Artificial intelligence chatbot research has focused on technical advances in natural language processing and validating the effectiveness of human-machine conversations in specific settings. However, real-world chat data remain proprietary and unexplored despite their growing popularity, and new analyses of chatbot uses and their effects on mitigating negative moods are urgently needed.

**Objective:**

In this study, we investigated whether and how artificial intelligence chatbots facilitate the expression of user emotions, specifically sadness and depression. We also examined cultural differences in the expression of depressive moods among users in Western and Eastern countries.

**Methods:**

This study used SimSimi, a global open-domain social chatbot, to analyze 152,783 conversation utterances containing the terms “depress” and “sad” in 3 Western countries (Canada, the United Kingdom, and the United States) and 5 Eastern countries (Indonesia, India, Malaysia, the Philippines, and Thailand). Study 1 reports new findings on the cultural differences in how people talk about depression and sadness to chatbots based on Linguistic Inquiry and Word Count and n-gram analyses. In study 2, we classified chat conversations into predefined topics using semisupervised classification techniques to better understand the types of depressive moods prevalent in chats. We then identified the distinguishing features of chat-based depressive discourse data and the disparity between Eastern and Western users.

**Results:**

Our data revealed intriguing cultural differences. Chatbot users in Eastern countries indicated stronger emotions about depression than users in Western countries (positive: *P*<.001; negative: *P*=.01); for example, Eastern users used more words associated with sadness (*P*=.01). However, Western users were more likely to share vulnerable topics such as mental health (*P*<.001), and this group also had a greater tendency to discuss sensitive topics such as swear words (*P*<.001) and death (*P*<.001). In addition, when talking to chatbots, people expressed their depressive moods differently than on other platforms. Users were more open to expressing emotional vulnerability related to depressive or sad moods to chatbots (74,045/148,590, 49.83%) than on social media (149/1978, 7.53%). Chatbot conversations tended not to broach topics that require social support from others, such as seeking advice on daily life difficulties, unlike on social media. However, chatbot users acted in anticipation of conversational agents that exhibit active listening skills and foster a safe space where they can openly share emotional states such as sadness or depression.

**Conclusions:**

The findings highlight the potential of chatbot-assisted mental health support, emphasizing the importance of continued technical and policy-wise efforts to improve chatbot interactions for those in need of emotional assistance. Our data indicate the possibility of chatbots providing helpful information about depressive moods, especially for users who have difficulty communicating emotions to other humans.

## Introduction

### Background

Chatbots are computer programs that simulate humanlike responses and interactively converse with users [1]. Advances in natural language processing (NLP) technology are leading to the generation of sophisticated responses that are increasingly natural and humanlike [2].

Chatbots have been shown to have a positive impact on mental health as they can help people manage feelings of depression, loneliness, and stress [3-6]. For instance, commercial chatbots such as Woebot [4], Wysa [7], Vivibot [8], and Tess [5] are aimed at alleviating mental health problems and have been effective in reducing anxiety and stress. Previous research on Wysa app users found that app use substantially reduced anxiety and depression symptoms during the COVID-19 pandemic [9]. College students who used Tess for 8 weeks reportedly had fewer anxiety symptoms [5].

Virtual assistants, including chatbots, have several advantages in assisting users when it comes to discussing sensitive topics. For instance, some people feel more comfortable disclosing depressive symptoms to virtual counselors [10]. Virtual agents provide anonymity and privacy, allowing users to express themselves without fear of being judged [6]. In addition, it is less challenging for users to talk to virtual agents than to other humans, such as friends, family, or therapists [3,6]. Finally, these systems are available at all times and are more convenient and immediate for users than seeking assistance through an appointment [3].

Although artificial intelligence (AI)–powered chatbots have potential mental health benefits, previous research has primarily concentrated on technical advances [11] or validating their efficacy in alleviating depressive symptoms and evaluating user engagement [12,13]. However, there is still a lack of research into real-world chat conversations related to depressive moods. Moreover, most previous research has been conducted in controlled laboratory settings, through self-reported surveys [3,4,10], or by analyzing depressive discourse in digital channels using social media data [14-17]. As a result, we currently lack real-world evidence on how users engage with emerging platforms such as chatbots. In addition, cultural differences have a significant impact on social behaviors, including the expression of emotions and discussion of mental health on social media [18]. Whether this is the case for AI-based chatbots has not been quantitatively assessed.

In this study, we analyzed real-world data from a commercial chatbot service to gain insights into the affective uses of this technology. This study investigated Eastern and Western chatbot users from different countries to examine how users express various affective states such as sadness and depression to anonymous conversational platforms. The data indicate that there are culturally specific differences in the expression of emotional states to chatbots and differences in what users express to a chatbot compared with on social media platforms.

We aimed to quantitatively examine how users disclose moods such as depression to chatbots during real-world use and understand the type of social support that users expect when expressing their emotions to such technology. We also wanted to test whether there are differences in sentiment and the topics that Western and Eastern chatbot users raise regarding depressive or sad moods. This second question would reveal whether chatbots may be culturally attuned to provide optimally beneficial mental health support.

To address these research questions, we carried out 2 analytical studies: studies 1 and 2. We analyzed a total of 152,783 chatbot-human interactions from SimSimi, a popular, globally used commercial chatbot service. The research goal was to identify emotional characteristics related to depression or sadness as well as cultural variations in these characteristics. We split the data into 5 Eastern (Indonesia, India, Malaysia, the Philippines, and Thailand) and 3 Western (Canada, the United Kingdom, and the United States) countries and used Linguistic Inquiry and Word Count (LIWC) [19] and n-gram techniques to compare cultural variations (study 1). We also used a semisupervised learning method to better classify chat topics and determine the most prevalent categories of depressive mood expressed in chatbot conversations. To contextualize our findings, we compared them with those of a study that looked at depressive discourse on a social media platform (Twitter, which was subsequently rebranded as X; study 2).

**Figure 1:**
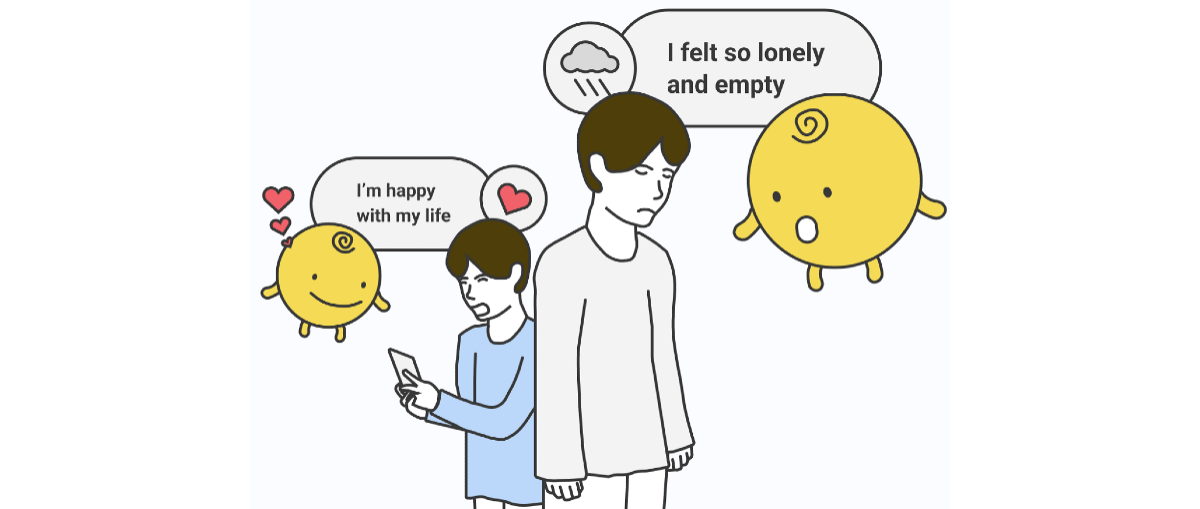
Users shared emotional messages, including depressive moods, with the chatbot, indicating the potential use of chatbots for emotional support.

This study found that individuals were more likely to express their negative self-perceptions and vulnerable emotions related to depressive or sad moods on chatbots than on social media (as illustrated in Figure 1). The semisupervised learning–based classification analysis revealed that Western users discussed their negative self-perceptions, whereas Eastern users expressed their current feelings more frequently to the chatbot. Eastern users expressed emotionally charged messages, both positive and negative, more often than Western users on chatbots.

This observation contrasts with earlier research that suggested that Eastern users tend to share positive emotions but avoid expressing negative emotions on social media [18]. Eastern users used more words associated with sadness on the chatbot. However, Western users used more words associated with vulnerable topics such as mental health and taboo topics such as sex and swear words. Chatbot conversations involved less seeking of feedback or emotional support from others compared with social media discourse. The data suggest that users expect chatbots to provide a safe space to express their emotions and advice or information to alleviate negative emotions. However, there were differing expectations regarding the role of social support aspects in chatbots between the Eastern and Western groups.

The results of this study provide substantial evidence that users express their depressive or sad emotions during interactions with an open-domain chatbot designed for small talk and entertainment. The findings highlight the potential for chatbot services to offer psychological support in times of need, and we propose several key design implications based on empirical evidence. We also discuss potential directions for future studies that could further expand our understanding of users’ needs across different cultures and languages.

### Related Research

#### Social Media Discourse on Depressive Moods

Social media has become a popular medium for people to express their emotions and discuss mental health. Social support and self-disclosure are 2 critical ways in which social media can help enhance mental health. Indeed, social media platforms have become spaces in which people share their experiences with mental illness. Previous research has shown that users seek support and learn more about their symptoms on platforms such as Twitter and Instagram [14,20,21]. Self-disclosure on social media has been argued to have a positive impact on mental health, with some studies referring to it as the *talking cure* because of its therapeutic and stress-relieving effects [17,22,23]. Venting negative emotions through self-disclosure has also been suggested to improve mental health according to the catharsis theory [24,25].

Despite the therapeutic advantages of self-disclosure on social media, many individuals prefer to talk about depressive moods anonymously because of social stigmas associated with depression [14]. In addition, openly discussing negative events on social media may be viewed as undesirable as it can elicit negative reactions from others [26]. As a result, people who are worried about social stigma are more likely to disclose sensitive information about their mood on anonymous platforms such as Twitter. Twitter’s pseudonym feature enables users to connect with others and discuss their mental health issues without the fear of being judged [16].

The distinct characteristics of web-based platforms can also affect how people talk about their depressive moods. For instance, Twitter is often used to share health information but not to find specific or high-quality information about treatment and diagnosis owing to its character limit [27]. In contrast, Reddit is often used to discuss experiences with mental illness and share specific health information; for example, Reddit users may seek a diagnosis or treatment [15]. In contrast, Facebook updates can raise concerns about privacy, prompting individuals to share more positive news and information [28].

Lachmar et al [16] investigated how individuals discuss their experiences of depression on Twitter. They identified 7 distinct themes related to users’ expressions of depressive moods by analyzing public discourse under the #MyDepressionLooksLike hashtag and performing a qualitative content analysis of approximately 2000 tweets. Although previous research has explored the analysis of depressive discourse on social media [14,16,21] and the categorization of various types of depressive discussions [16], there is a notable gap in research concerning the identification and categorization of the types of depressive discourse found in emerging chatbot platforms. Consequently, it remains uncertain whether users’ self-disclosure behavior and the effectiveness of chatbots as sources of social support differ from those on social media. To address this gap, this study used a deep learning–based semisupervised algorithm to uncover themes related to the expression of depressive moods within chatbot interactions. This comprehensive analysis was conducted using large-scale data sets. This study represents a significant advancement in analytical efficiency compared with previous research, which primarily relied on qualitative analyses of relatively modest data sets. In this study, we examined how users disclose personal information when interacting with chatbots.

#### Self-Disclosure to the Conversational AI

Previous research suggests that conversational AI can have positive effects on mental health and well-being. For instance, conversational AI platforms (or chatbots) have been reported to reduce loneliness and provide a safe space for discussing sensitive issues owing to their perceived nonjudgmental nature [6]. The recognition that conversational AI is not human can increase both user trust and comfort, leading to greater self-disclosure without fear of being judged [6]. A study conducted by Lucas et al [10] showed that people were more willing to share personal information with machine-operated agents than with human-operated agents. In a workplace context, employees preferred AI counselors over human counselors to disclose their stress or anxiety [29].

In addition, cognitive behavioral therapy conversational agent apps have been shown to effectively reduce depressive symptoms. In one study, the anxiety and depressive moods of the group that received self-help through a conversational AI app were significantly lower than those of the control group [4]. Another study focused on college students and found that anxiety was significantly lower in the experimental group, which used a cognitive behavioral therapy chatbot app designed to alleviate depressive moods for 8 weeks, compared with the control group [5].

Despite the promising results of previous studies [4,5,10] on chatbots and mental health, these studies are not without limitations. Many studies [4,5,7] have focused on the effectiveness of commercial chatbot apps designed to alleviate depressive symptoms or relied on data collected from prototype chatbots in experimental settings [10]. Furthermore, only a few studies have examined real-world interactions between users and chatbots. A recent study investigated conversations between users and a commercially available social chatbot, but it only focused on pandemic-related chat conversations [30]. Through a comprehensive analysis of real-world chatbot interactions, our study validated the occurrence of self-disclosure behaviors among chatbot users experiencing depressive moods, aligning with observations made in controlled experimental settings. In addition, by using a substantial data set that encompasses diverse cultural contexts, this research extends previous studies by investigating the use of chatbots as a means to alleviate depressive moods within the context of everyday life.

#### Cross-Cultural Differences in Depressive Discourse

Differences in culture [31], race [32], class [33], and gender [34] have been acknowledged to have a significant impact on how individuals behave on the web, particularly in terms of expressing their mental health, disclosing information, and displaying emotions on social media. Previous research has revealed that cultural variations exist between Eastern and Western countries in how people with mental illnesses use web-based mental health forums [35]. Pendse et al [35] analyzed platform use across 3 dimensions—identity, language use, and support behaviors—and found that Eastern users are more likely to mention their country of origin, use less clinical language to talk about their mental distress, and prefer support from individuals from the same country.

A study revealed that individuals in Western countries experience more loneliness and lack of confidence [18], whereas individuals in Eastern countries such as India [31], Malaysia [36], and the Philippines [37] tend to show physical manifestations of their mental distress. In addition, Wang et al [38] discovered that within traditional Chinese society, individuals typically hesitate to openly convey their emotional distress. When facing difficulties in their daily functioning that could potentially be linked to psychological distress, they tend to ascribe these challenges to physical or external factors. These findings demonstrate cultural distinctions in how people use social media for mental health, underscoring the need to consider these differences when creating a web-based platform for mental health support. In alignment with previous research outcomes, this study explored the validity of cultural disparities in the manifestation of depressive mood expressions as observed in social media or web-based forums, extending this examination to encompass chatbot platforms.

## Data Methodology

### SimSimi Chatbot

**Figure 2:**
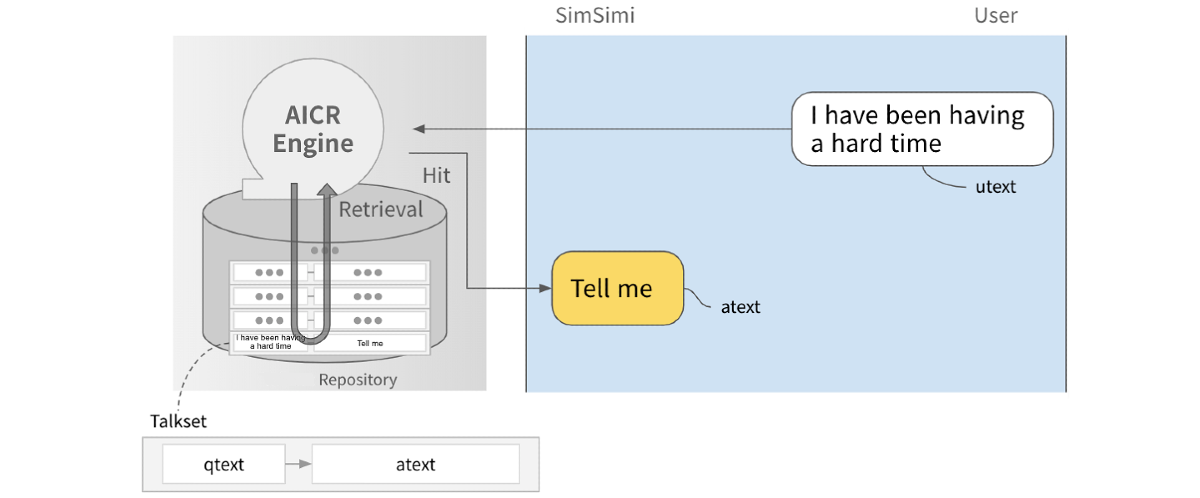
Snapshot of the service and the response-generation mechanism. Diagram of SimSimi application user interface and an example of a session (left) and the architecture of the SimSimi service (right). AICR: Artificial Intelligence for Chatting Robot.

To analyze the interaction between individuals and chatbots in terms of depressive moods, we analyzed user conversations with SimSimi, one of the world’s most widely used chatbot services. SimSimi is a chatbot designed to entertain users through social interaction. It was launched in 2002 and has served 400 million users in 111 languages and up to 200 million daily chat utterances. Utterances include anything that the user inputs into the chatbot. SimSimi also includes a teaching function in which users are guided on how to answer a specific question. The user-generated responses from this function are added to the main database. The service then searches this database to generate the most appropriate response for future user utterances (Figure 2). At the time of analysis, SimSimi’s database contained 114.6 million conversation pairs.

There are several benefits to studying this particular chatbot. First, SimSimi is different from other chatbots such as Woebot and Wysa, which are designed to treat depression. SimSimi is an open-domain chat service, which means that it can talk about anything. This makes SimSimi more similar to real-world conversations, which is important for understanding how people seek social support.

Second, SimSimi is available on the web and as a mobile app, which makes it easy for people to use. This ensures that SimSimi will have a large number of users, which is important for data collection. Third, SimSimi has users from all over the world, which allows us to study cultural differences by categorizing the data by region.

### Data Sets

SimSimi Inc is allowed to use content posted by users or associated with SimSimi services under their terms and conditions. This license is nonexclusive, transferable, relicensable, royalty free, and applied worldwide [30]. SimSimi Inc reports novel findings from aggregate data on its research blog, informing users about data use and providing insights into the service. SimSimi Inc also obtains user consent to access data via the terms and conditions.

Researchers were given the following data: the time of the chat, unique user ID generated by the app, country, user query, and chatbot response to the user query. However, no personally identifiable information (PII) was included in the data. SimSimi does not collect or store PII such as username, gender, age, phone number, social security number, or address. SimSimi protects user privacy even when users reveal PII during their conversation using internal text-filtering methods (eg, replacing consecutive numbers with strings of asterisks). When users install the SimSimi app, geolocation information is obtained based on the time zone settings of their devices, and this time zone information is assigned to a specific country based on the tz database [39].

The anonymized data we received from SimSimi were collected from May 1, 2016, to December 31, 2020. The data included sentences containing specific keywords related to depressive moods. These keywords were [40] “depress,” “distress,” “dejected,” “gloomy,” “cheerless,” “sad,” “feeling low,” “hate myself,” and “ashamed of myself” (Table 1).

We emphasize that our focus was not on people clinically diagnosed with depression or other mental illnesses. Instead, our target data aimed to represent general internet users who happen to discuss depressive or sad moods. To select target users, we began by referring to previous studies on the topic of cross-cultural differences in web-based forums and social media [31,35]. Unlike research primarily centered on Western contexts, these studies explored how users in non-Western countries such as India, Malaysia, the Philippines, and South Africa expressed depressive moods. In particular, a study conducted by Pendse et al [35] revealed that web-based users in India, Malaysia, and the Philippines shared similar cultural contexts when expressing depressive emotions. To further explore this, they compared the data of users from these 3 countries with those of users from the United States, the United Kingdom, and Canada. When selecting the target countries, they considered not only the cultural context but also the countries with the highest user populations on the platform.

On the basis of the review of these studies and criteria, we identified the following 8 countries as primary research targets for this study: Canada, Indonesia, India, Malaysia, the Philippines, Thailand, the United Kingdom, and the United States. These selections were also made by considering the countries with the highest number of conversation sets involving SimSimi users. As there may be cultural differences in how people express their emotions, we first grouped the data by country of origin. Malaysia, the Philippines, India, Indonesia, and Thailand represented Eastern cultures, and Canada, the United Kingdom, and the United States represented Western cultures. From here on, we will refer to these cultural groups as Eastern and Western users, respectively. We gathered data from 96,197 and 56,586 conversations for the Eastern and Western countries, respectively.

For study 2, all 152,783 data sets were examined to categorize different types of discourses related to depression. However, for study 1, we focused on the top 5 countries with the highest number of user-chatbot interactions (Malaysia, the Philippines, Canada, the United Kingdom, and the United States) to compare the hourly chat frequency and quantified linguistic traits of depressive conversations between the 2 regions. We observed that there were fewer depressive mood–related conversations among users in Indonesia, India, and Thailand compared with the other 5 countries studied. To prevent one user’s conversation from dominating the data set, we randomly selected one statement from each user. The Eastern and Western group each consisted of 21,156 users who made utterances about depressive moods. Table 1 provides an overview of the data used in the study.

**Table 1:** The number of data points extracted using keywords related to depressed or ad and happy or excited states.

Keywords	Data points (Eastern countries), n	Data points (Western countries), n	Analysis method	Country	Keywords
Depressed or sad	92,955	55,635	Classifications	Malaysia, the Philippines, India, Indonesia, Thailand, Canada, United Kingdom, and United States	“Depress,” “distress,” “dejected,” “gloomy,” “cheerless,” “sad,” “feeling low,” “hate myself,” and “ashamed of myself”
Depressed or sad	21,156	21,156	Temporal analysis, LIWC^a^, and n-gram	Malaysia, the Philippines, Canada, United Kingdom, and United States	“Depress,” “distress,” “dejected,” “gloomy,” “cheerless,” “sad,” “feeling low,” “hate myself,” and “ashamed of myself”
Happy or excited	12,705	12,705	Temporal analysis	Malaysia, the Philippines, Canada, United Kingdom, and United States	“Elated,” “overjoyed,” “enjoy,” “excited,” “proud,” “joyful,” “happy,” “feel blessed,” “blessed,” “amazing,” “wonderful,” “excellent,” “delighted,” and “enthusiastic”

^a^LIWC: Linguistic Inquiry and Word Count.

### Ethical Considerations

This study was conducted in collaboration with SimSimi Inc with strict privacy considerations in place. The Korean Advanced Institute of Science and Technology Institutional Review Board reviewed the data collection plan and study methodology and waived the need for user-informed consent for this study. The study team adhered to institutional review board protocols (Korean Advanced Institute of Science and Technology IRB-21-494). We extracted and used only sentences containing specific keywords relevant to this study. We did not analyze any aggregated data at the individual user level. When quoting users’ utterances, we paraphrased rather than using direct quotes from the chat [41]. Finally, this study was conducted in collaboration with health care professionals.

### Research Overview

**Figure 3:**
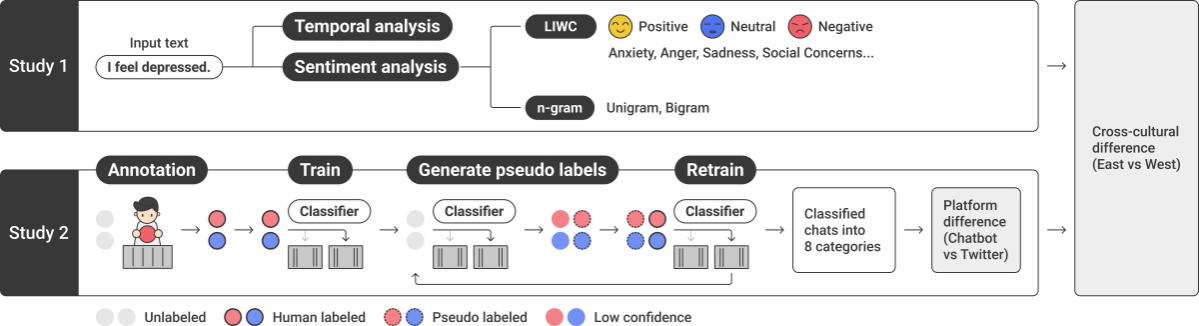
Overview of study 1 and study 2. LIWC: Linguistic Inquiry and Word Count.

Figure 3 illustrates the research methodology. The objective of study 1 was to gain in-depth and comprehensive insights into the data. Initially, we examined the frequency of relevant conversations over time to determine the prevalence and extent of depressive or sad emotions among users. To quantify the cultural and linguistic differences in topic and sentiment, we used NLP tools such as LIWC and n-gram.

In study 2, we classified chat conversations into predefined topics using human-annotated data and semisupervised classification techniques to gain a better understanding of the type of depressive mood prevalent in chatbots. We then identified the distinguishing features of chat-based depressive or sad discourse by comparing the categorical frequency in chatbot data and social media posts in the Eastern and Western groups.

## Study 1: Cross-Cultural Differences

### Methods

#### Step 1: Temporal Analysis

We used log data to determine when users expressed depressive or sad emotions to the chatbot. The Russell circumplex model of affect [42] suggests that “happy” and “excited” are polar opposites of “depressed” and “sad.” Therefore, we compared the hourly chat frequency of these emotions and examined how chat patterns varied over the course of a day for Eastern and Western users.

We used the Python *pandas* and *matplotlib* libraries (Python Software Foundation) for data analysis and visualization. The relative chat frequency was calculated by dividing the number of chats per hour by the total number of chats in that region. The time stamp was matched to the standard time zone of each country.

#### Step 2: Quantifying Linguistic Traits of Depressive Discourse

The way individuals express themselves through language can reveal their psychological state [43]. We used LIWC [19] to analyze the language in a quantifiable and objective manner, categorizing words into meaningful psychological and social categories. The study focused on 6 linguistic categories, and the patterns for Eastern and Western users were compared using Welch *t* tests (2-tailed).

The following six measures were derived from LIWC: (1) affective processes (words representing the 6 measures of emotional expression: positive, negative, anxiety, anger, sadness, and swear words), (2) biological processes (words reflecting body-oriented or biological processes such as physical, health, mental health, sexual, or death), (3) social or personal concerns (words related to family, friends, home, and work), (4) time orientation (words related to time orientation in general, such as a past, present, and future focus), (5) perceptual processes (words representing multiple sensory and perceptual dimensions associated with the senses, such as *feel*, *look*, and *heard*), and (6) interpersonal awareness and focus (first-, second-, and third-person pronouns).

In addition, the top 10 unigrams and bigrams were examined, with stop words, single-letter words, and special characters removed using Python’s Natural Language Toolkit and *sklearn* libraries for preprocessing. To ensure a balanced comparison, we matched the data size for the LIWC and n-gram analyses to 21,156 depressive or sad utterances for both the Eastern and Western groups (as shown in Table 1).

### Results

#### Chat Distributions Over Time

**Figure 4:**
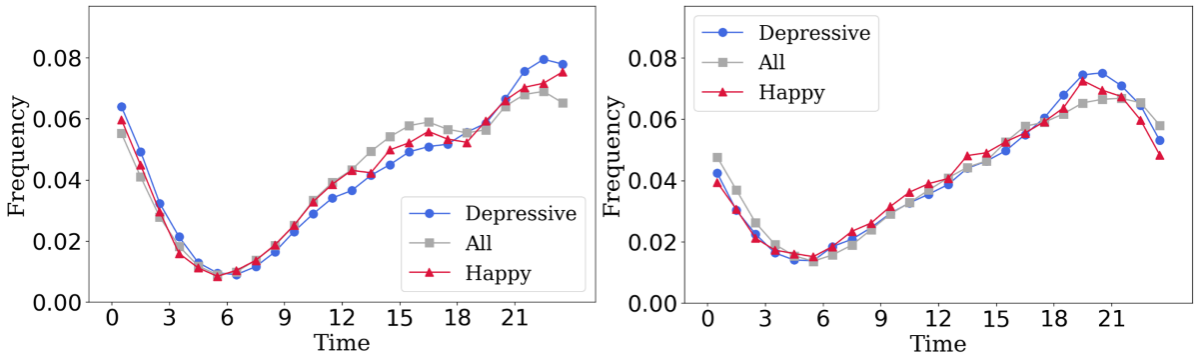
The relative hourly chat frequency by emotional expression. The graphs show the chat frequency by topic in Eastern (left graph) and Western (right graph) users.

Figure 4 compares the relative frequencies of 3 chat types: depressive (containing depression- or sadness-related keywords), happy (containing happiness- or excitement-related keywords), and general (indicating all SimSimi utterances in the same period). SimSimi Inc provided the statistics for the general utterance trends.

The plot demonstrates that depressive emotions varied throughout the day. However, depressive conversations were more frequent late at night rather than during the day. Daytime utterances were more likely to include happy or excited emotions than depressed emotions.

The chat emotions differed by culture. In contrast to the general trend, depressive emotions occurred late in the day for both Western and Eastern users, although their peak times differed. Depressive emotions were most prevalent between 7 PM and 8 PM for Western users and between 9 PM and 10 PM for Eastern users. In Eastern countries, late chats persisted, as did significantly higher levels of depressed emotions during these hours compared with Western users.

General conversation topics were more common between 10 AM and 6 PM for Eastern users, whereas depressed or happy chats increased sharply after 7 PM. The difference in chat frequency by topic and time for the West, in contrast, followed a similar pattern to that for Eastern users but was not as significant (Figure 4).

#### Sentiment Difference by Geographic Region: LIWC and N-Gram

Table 2 shows the results of the LIWC analysis. In total, 6 subcategories were used. First, Western and Eastern user data showed a clear difference in the use of words related to affective processes. LIWC sentiment analysis revealed cultural differences in terms of positive sentiment (t_38,680_=8.36; *P*<.001), negative sentiment (t_41,417_=2.55; *P*=.01), and sad emotion scores (t_41,402_=2.48; *P=*.01). Eastern users were more positive (Eastern: mean 0.81, SD 3.96; Western: mean 0.52, SD 3.04) and more negative (Eastern: mean 29.68, SD 20.76; Western: mean 29.17, SD 20.40) in their use of affective terms than Western users. This group was more likely to use words associated with sadness (Eastern: mean 28.61, SD 21.32; Western: mean 28.10, SD 20.83). However, when it came to engaging in depressive or sad chats, Western users used a larger average number of swear words than Eastern users when expressing their feelings (t_35,782_=−8.17; *P*<.001; Eastern: mean 0.19, SD 2.15; Western: mean 0.41, SD 3.31).

**Table 2:** Welch *t* test results examining the quantified linguistic differences through Linguistic Inquiry and Word Count (LIWC) analysis in the depressive discourse of Eastern and Western chatbot users.

LIWC category			*t* test (*df*)		*P* value		Eastern users, mean (SD)		Western users, mean (SD)
**Affective processes: words of emotional expression**									
	Emo_pos^a^	8.36 (38,680)		<.001		0.81 (3.96)		0.52 (3.04)	
	Emo_neg^b^	2.55 (41,417)		.01		29.68 (20.76)		29.17 (20.4)	
	Emo_anx^c^	−0.89 (41,161)		.38		0.08 (1.22)		0.09 (1.34)	
	Emo_anger^d^	1.68 (41,336)		.09		0.75 (4.44)		0.68 (4.26)	
	Emo_sad^e^	2.48 (41,402)		.01		28.61 (21.32)		28.1 (20.83)	
	Swear	−8.17 (35,782)		<.001		0.19 (2.15)		0.41 (3.31)	
**Biological processes: body-oriented words or terms linked to biological processes**									
	Physical	−17.16 (38,642)		<.001		2.41 (9.15)		4.22 (12.19)	
	Health	−14.52 (38,743)		<.001		2.03 (8.78)		3.5 (11.64)	
	Mental	−14.48 (38,665)		<.001		1.94 (8.68)		3.39 (11.56)	
	Sexual	−5.83 (34,934)		<.001		0.05 (0.94)		0.12 (1.51)	
	Death	−4.52 (39,108)		<.001		0.08 (1.13)		0.14 (1.47)	
**Social and personal concerns: words related to social functions**									
	Family	−1.2 (41,369)		.23		0.23 (2.17)		0.26 (2.29)	
	Friend	−2.2 (41,275)		.03		0.18 (1.61)		0.22 (1.75)	
	Home	−4 (36,882)		<.001		0.03 (0.64)		0.06 (0.93)	
	Work	−2.19 (39,489)		.03		0.09 (1.07)		0.11 (1.35)	
**Time orientation: words related to past, present, or future focus**									
	Past focus	−8.55 (40,306)		<.001		0.86 (4.01)		1.24 (4.81)	
	Present focus	36.25 (41,120)		<.001		19.45 (18.67)		13.05 (17.25)	
	Future focus	−0.51 (41,424)		.61		0.39 (2.53)		0.4 (2.5)	
**Perceptual processes: words of sensory and perceptual dimensions**									
	Visual	−6.21 (36,391)		<.001		0.13 (1.53)		0.25 (2.29)	
	Auditory	1.42 (41,360)		.16		0.19 (2.08)		0.16 (2.01)	
	Feel	6.21 (40,875)		<.001		1.86 (6.48)		1.48 (5.82)	
**Interpersonal awareness and focus: words related to singular and plural pronouns**									
	First person	3.11 (41,085)		.002		20.61 (18.49)		20.06 (17)	
	Second person	−5.6 (41,233)		<.001		3.43 (8.15)		3.9 (8.86)	
	Third person	0.13 (41,441)		.90		0.26 (1.97)		0.26 (1.96)	

^a^Emo_pos: positive emotion.

^b^Emo_neg: negative emotion.

^c^Emo_anx: anxiety emotion.

^d^Emo_anger: anger emotion.

^e^Emo_sad: sad emotion.

When comparing depressive discourses, Western users used words related to personal concern subtopics more frequently than Eastern users. The average percentage of words related to biological processes in all chats was higher in Western users than in Eastern users, particularly in conversations related to depressive moods. This group mentioned physical (t_38,642_=−17.16; *P*<.001; Eastern: mean 2.41, SD 9.15; Western: mean 4.22, SD 12.19) and health (t_38,743_=−14.52; *P*<.001; Eastern: mean 2.03, SD 8.78; Western: mean 3.50, SD 11.64) topics far more frequently than Eastern users, as well as topics related to mental health (t_38,665_=−14.48; *P*<.001; Eastern: mean 1.94, SD 8.68; Western: mean 3.39, SD 11.56). The average percentage of sexual (t_34,934_=−5.83; *P*<.001; Eastern: mean 0.05, SD 0.94; Western: mean 0.12, SD 1.51) and death-related (t_39,108_=−4.52; *P*<.001; Eastern: mean 0.08, SD 1.13; Western: mean 0.14, SD 1.47) words being used in depressive mood conversations was also higher in the Western group. Furthermore, the use of vocabulary related to home, friends, and work was also statistically significantly more prevalent in Western users than in Eastern users (Table 2).

The difference in time orientation terms used by each group was also noteworthy. Eastern users tended to use the present tense more frequently in chats (t_41,120_=36.25; *P*<.001; Eastern: mean 19.45, SD 18.67; Western: mean 13.05, SD 17.25), whereas Western users tended to favor the past tense (t_40,306_=−8.55; *P*<.001; Eastern: mean 4.01, SD 1.24; Western: mean 1.24, SD 4.81). In addition, Eastern users used words in the feeling category more frequently during chats than Western users, including “pain,” “hurt,” “sore,” and “feelings” (t_40,875_=6.21; *P*<.001; Eastern: mean 1.86, SD 6.48; Western: mean 1.48, SD 5.82). However, compared with users in the East, Western users used more terms related to visual perception (t_36,391_=−6.21; *P*<.001; Eastern: mean 0.13, SD 1.53; Western: mean 0.25, SD 2.29).

The frequency of use of first-person and second-person pronouns also differed significantly between Eastern and Western users (first person: t_41,085_=3.11 and *P*=.002; second person: t_41,233_=−5.6 and *P*<.001). Although second-person pronouns were less common (Eastern: mean 3.43, SD 8.15; Western: mean 3.90, SD 8.86), first-person pronouns were more common in Eastern users’ chats than in Western users’ chats (Eastern: mean 20.61, SD 18.49; Western: mean 20.06, SD 17.00).

Table 3 displays the unigrams and bigrams of the Eastern and Western data. Eastern users used the phrase “feel,” which is associated with the perceptual process, more frequently. Furthermore, as evidenced by the bigram results, phrases such as “right now” and “feel sad” were commonly used to express current depressive moods among Eastern users.

**Table 3:** Top 10 unigram and bigram results comparing users in Eastern and Western countries. The numbers in parentheses indicate the number of counts.

	Eastern countries	Western countries
Unigram	“Sad” (0.14), “im” (0.05), “you” (0.04), “so” (0.03), “lonely” (0.03), “me” (0.02), “to” (0.02), “feel” (0.01)^a^, “and” (0.01), and “that“ (0.01)	“Sad” (0.12), “you” (0.04), “lonely” (0.03), “and” (0.02), “to” (0.02), “me” (0.02), “so” (0.02), “im” (0.02), “depressed” (0.02), and “it” (0.01)
Bigram	“im sad” (0.03), “so sad” (0.02), “sad im” (0.02), “sad sad” (0.01), “you sad” (0.01), “im so” (0.01), “right now” (0.01), “me sad” (0.01), “feel sad” (0.01), and “hate myself” (0.01)	“Sad sad” (0.02), “im sad” (0.01), “are you” (0.01), “so sad” (0.01), “you sad” (0.01), “me sad” (0.01), “sad im” (0.01), “am sad” (0.01), “hate myself” (0.01), and “be sad” (0.01)

### Discussion

#### Differences in Time Expressing Depressive Moods via Chats Between the East and the West

Chats containing depressive emotions were more prevalent after 7 PM in both Eastern and Western users. In particular, Eastern users had a higher frequency of depressive chats from late night until dawn compared with Western users. Previous research has shown that bedtime is the most common time for depressive chats with a chatbot in both cultural groups, with Eastern users having later bedtimes than Western users [44]. The average bedtime in Canada, the United Kingdom, and the United States is approximately 11 PM, whereas bedtime in Malaysia is approximately 12:30 AM [45,46]. As most of SimSimi interactions were via mobile devices (>99.5%), similar to depressive interactions on social media, it appears that users express their depressive feelings to chatbots around bedtime [46]. The influence of time on changes in chat topics was slightly more significant for Eastern users.

#### Sentiment Differences in Chats in Eastern and Western Countries

According to the LIWC analysis, Eastern users showed a greater tendency to share emotionally charged messages compared with Western users when discussing depressive moods. Eastern users exhibited a higher average percentage of affective process–related words, including those expressing positive sentiments, negative sentiments, and sadness, indicating that they expressed more negative and positive sentiments (eg, “im .....so.....sad....sob sob......T_T”). These results contrast with previous research [18] that used the LIWC software to explore cultural distinctions in depressive moods on Twitter. They examined the Twitter feeds of those who had depression and found that non-Western users (eg, from India and South Africa) exhibited more positive affect and less negative affect, anger, anxiety, and sadness compared with Western users (eg, from the United States and the United Kingdom). However, the study revealed that Eastern users expressed both positive and negative sentiments more frequently when discussing depression.

East Asian cultures are often seen as collectivist, with cooperative in-group relationships and a focus on social harmony [47]. This is demonstrated by research showing that East Asian populations tend to suppress their emotions in interpersonal relationships to avoid confrontation and maintain group harmony [48]. However, our analysis of chatbot conversations suggests that Eastern users are more willing to express not only positive but also negative emotions (eg, “im happy in the outside but deep in the inside im sad:)”) and perceptual feelings to an anonymous chatbot platform, differing from previous studies examining social media.

Chatbot conversations revealed that Eastern users use more perception-related words, specifically in the feeling category (eg, “pain,” “hurt,” “sore,” and “feelings”), and frequently use first-person pronouns when discussing their depressive moods. In addition, their chats tend to be more present focused compared with those of Western users, indicating that they are more likely to openly express their current emotional state. This pattern is further supported by the results of unigram and bigram analyses of chatbot conversations, which show that Eastern users use more terms related to the expression of their depressed mood, such as “feel,” “feel sad,” and “right now,” than Western users.

Consistent with previous research on depressive moods on Twitter [18], this study found that Western users are more likely to discuss functioning, including social concerns, physical health, and mental health–related topics, in chatbot conversations than Eastern users (eg, “I’m depressed loss of appetite”). They are also more likely to talk about sensitive topics such as death (eg, “I’m depressed and suicidal”) and sexuality and use more profanity with chatbots (as shown in Table 2). Furthermore, Western users show a greater interest in people and everyday issues such as home, friends, and work (eg, “my friends make me sad”), indicating that they are more focused on discussing daily life issues and the people around them than on simply expressing negative emotions. These findings indicate that Western users tend to express their physical and mental illness experiences more openly on the web, whereas Eastern users tend to suppress expressions of mental illness and instead discuss their feelings related to depressive moods.

To summarize, it is clear that there are cultural variations in how chatbot users express depressive moods. Eastern users tend to share more emotionally intense messages compared with their Western counterparts. The pattern observed in Eastern users is different from that observed in previous studies examining depressive mood expressions on Twitter [16]. However, given the nascent state of research on depressed mood expression in chatbots, further investigation is necessary to validate these results and investigate linguistic and topic differences among a more diverse range of cultural groups.

## Study 2: Depressive Moods on Chatbots

### Overview

Using a machine learning–based classifier, study 2 analyzed extensive chatbot data and classified prevalent topics related to depressive moods into pre-established categories (Table 4). The overall methodology is presented in Figure 3.

**Table 4:** A total of 7 depressive discourse categories, descriptions, and representative chat examples.

Category	Description [16]	Chat example
Dysfunctional thoughts	Thoughts about the self that are negative and hopeless	“I hate everything, I hate life, I hate myself”
Lifestyle challenges	Difficulty with eating, sleeping, and daily tasks	“I can’t sleep at night because I’m so sad and stressed”
Social struggles	Struggling with social relationships and loneliness	“I tried telling her about my depression and she just yelled at me and called me crazy.”
Hiding behind a mask	Pretending to be OK in front of others	“yeah...I’m just a bit depressed...I’ll be okay.”
Apathy and sadness	Expressions of sadness and emptiness	“I’m sad and crying now”
Suicidal thoughts and behaviors	Descriptions of self-harm and thoughts of death	“I want to die but I don’t kill my self because I don’t like pain”
Seeking relief	Both positive and negative means to alleviate depression	“can you give a advice or heal my sad”

### Methods

#### Overview

Semisupervised learning is known to be effective when labeled data are scarce whereas the amount of unlabeled data is large [49]. In the case of chatbot data involving conversations in depressive moods, the available labeled data are quite limited. Moreover, the task of labeling nearly 150,000 chats expressing depressive moods in chatbot data is both time-consuming and expensive. As a result, to efficiently and automatically classify substantial volumes of data, instead of annotating the entire data set, we adopted a strategy of randomly selecting approximately 1% of the data and labeling those samples. Subsequently, we used this 1% of labeled data to implement a semisupervised learning framework.

#### Data Annotation

To create a topic classifier, we manually labeled recurring themes in a random sample of 1500 utterances from depressed or sad chats. We borrowed the classification themes from previous research on Twitter on depressive emotions [16], which identified seven major categories: (1) dysfunctional thoughts, (2) lifestyle challenges, (3) social struggles, (4) hiding behind a mask, (5) apathy and sadness, (6) suicidal thoughts and behaviors (self-harm or death), and (7) seeking relief. Information that did not fit into any of these 7 categories was classified as (8) etc. Although there have been many previous studies on depressive categories expressed in various forms of user-generated content on the internet, we refer to the study that used Twitter data as Twitter is a short text–based platform similar to a chatbot. Table 4 shows the categories and matched examples from the chatbot data.

In total, 2 health care researchers and 2 data science researchers labeled the data. Before annotating the data, the definitions of the aforementioned 8 categories were shared with the researchers. Over 2 weeks, each researcher independently classified 1500 utterances into 8 categories. Following the collection of labeled data, data on which there was disagreement among at least 3 out of 4 labelers were discarded, totaling 1348 utterances. The Fleiss κ value of 0.85 for the 4 raters indicates high agreement.

#### Feature Extraction From Annotated Data and Classification Using Semisupervised Learning

##### Overview

We compared the performances of 5 popular classification models on labeled data to find the best model. The models we used were extreme gradient boosting (XGBoost), random forest, multilayer perceptron, naive Bayes, and support vector machine. We split the data into 80% training data and 20% testing data. We extracted features from the data using bag of words (BoW), part of speech (PoS), and the Bidirectional Encoder Representations from Transformers (BERT) language model [50]. We extracted BoW and PoS values using standard preprocessing, which removed URLs and stop words.

##### BoW Method

BoW is a simple but effective method for representing text. It counts the number of times each word appears in a document. BoW has been used to identify behavioral differences in vocabulary use patterns between individuals with and without depression. Several previous studies have used BoW to identify depressive moods or symptoms in the text [51-54]. To use BoW as a feature, we constructed a vocabulary set from the entire data set and excluded words that appeared <5 times. Each instance was converted into a multihot vector with a vocabulary size of 6027 using this vocabulary set.

##### PoS Method

PoS tagging identifies the PoS of each word in a sentence. PoS tags are used to represent grammatical patterns as a feature of sentence structure. Therefore, PoS tagging can be a useful tool for identifying the grammatical patterns of individuals with depression. Previous research [54] has shown that the grammatical patterns of Twitter users with depression differ from those of general Twitter users. The study found that individuals with depression were more likely to use first-person pronouns (eg, “I,” “me,” and “mine”) and negative emotion words (eg, “sad,” “lonely,” and “depressed”). For this analysis, we used Python’s *Spacy* package to identify the PoS tags of each word in a sentence. The total number of PoS tags was 46, and the frequency of each tag was counted.

##### Sentence Representation From Language Model

The rise of deep learning methods has led to the use of general-purpose pretrained language models in many NLP tasks. Language models such as BERT [50] have demonstrated significant performance improvements in various NLP tasks, including the identification of mental disorders in user-written texts [55]. In this experiment, we used BERT to extract the sentence representation of each chat instance. We used the Hugging Face BERT-base-uncased model to extract features from each chat instance [56]. Each chat instance was then converted into a 768-dimensional vector. By concatenating the 3 types of features, we generated 6841-dimensional vectors as input features (BoW=6027; PoS=46; BERT=768).

After selecting the best-performing model out of the 5 classifiers, we used it in a semisupervised learning procedure to classify the chatbot data into 8 predefined categories. We then analyzed the distribution of each category in the final classification data comparing Western and Eastern users.

Finally, we compared our chatbot-based analysis of commonly observed depressive or sad discourse categories to the commonly observed categories previously uncovered in Twitter data [16].

Table 5 shows the classification performance of the 5 models—XGBoost, random forest, multilayer perceptron, naive Bayes, and support vector machine—on labeled data. The multilayer perceptron classifier showed the best performance in all 5 evaluation indicators (accuracy=0.64; area under the receiver operating characteristic curve=0.86; macro–*F*_1_-score=0.60; precision=0.62; recall=0.59).

**Table 5:** The performance of supervised models on labeled data.

Method	Accuracy	AUROC^a^	*F*_1_-score	Precision	Recall
SVM^b^	0.46	0.84	0.27	0.42	0.29
Random forest	0.44	0.75	0.34	0.46	0.33
Naive Bayes	0.35	0.64	0.32	0.34	0.35
XGBoost^c^	0.59	0.85	0.54	0.56	0.53
Multilayer perceptron	0.64	0.86	0.6	0.62	0.59

^a^AUROC: area under the receiver operating characteristic curve.

^b^SVM: support vector machine.

^c^XGBoost: extreme gradient boosting.

Trained models can be used to make predictions for unlabeled data sets. The higher the prediction confidence, the better the model’s performance. For example, in a classification task, the test accuracy increases significantly when the confidence in the predicted label is above a certain level. We applied a general semisupervised framework that uses high-confidence prediction results as pseudo labels. In semisupervised learning, we used the multilayer perceptron architecture with the aforementioned classifiers. The semisupervised framework was performed through the following steps: (1) train a multilayer perceptron with labeled data, (2) use the model trained in step 1 to generate pseudo labels from unlabeled data whose predicted probability threshold is >0.9, (3) retrain the classifier with the labeled and pseudo labeled data from step 2, and (4) repeat steps 2 and 3. Tables 5 and 6 show the performance of the supervised and semisupervised models on the data. After semisupervised learning, the evaluation results for labeled test data with a threshold of ≥0.9 are reported in parentheses in Table 6.

We used a large unlabeled data set. We split the data set into 2 groups based on the user-chosen information: Eastern and Western users. The Eastern user group had 96,197 data points, whereas the Western user group had 56,586 data points (Table 1).

**Table 6:** The performance of semisupervised models on labeled data. Evaluation results with high confidence are reported in parentheses.

Method	Accuracy	AUROC^a^	*F*_1_-score	Precision	Recall
Eastern MLP^b^	0.63 (0.68)	0.87 (0.88)	0.59 (0.62)	0.61 (0.62)	0.59 (0.64)
Western MLP	0.63 (0.66)	0.86 (0.88)	0.56 (0.62)	0.57 (0.65)	0.57 (0.62)

^a^AUROC: area under the receiver operating characteristic curve.

^b^MLP: multilayer perceptron.

### Results

#### Classification Results of a Semisupervised Model for the Depressive Mood Category in Chatbots

**Figure 5:**
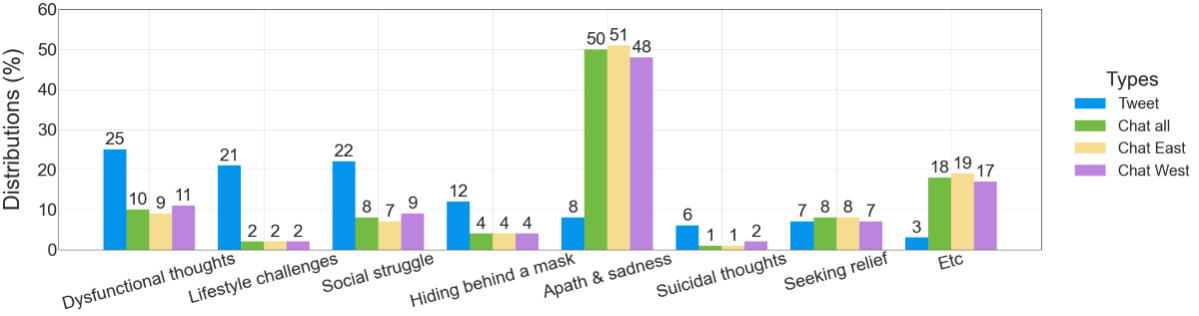
Distribution of depressive discourse in chatbots and tweets compared by category.

We used the prediction results with high confidence from the trained classifier in the previous section to investigate the differences in the distribution of pseudo labels according to predefined categories for the Western and Eastern data sets (Figure 5).

Apathy and sadness were the most common topics of conversation for both Western and Eastern chatbot users (Figure 5). More than half (74,045/148,590, 49.83%) of all conversations involved users expressing feelings of loneliness or sadness (eg, “I’m sad and crying now”). The next most common category, excluding the *etc* category, was dysfunctional thoughts (14,385/148,590, 9.68%), which refers to negative self-evaluation (eg, “I hate everything, I hate life, I hate myself”). The third most common category of conversations involved social struggles caused by depression. This category included conversations about how depression makes it difficult to function in daily life, such as “I tried telling her about my depression and she just yelled at me and called me crazy.” The fourth most common category was seeking relief from depression. This included conversations about how to cope with depressive symptoms or asking for help, such as “I have depression and anxiety, what should I do about this?” Each of these categories accounted for 8% (social struggle: 11,705/148,590, 7.88%; seeking relief: 11,451/148,590, 7.71%) of all depressive chats.

The *etc* category accounted for 18.24% (27,101/148,590) of all conversations. In this category, many chat users regarded the chatbot as a social actor and empathized with the chatbot’s answers rather than providing utterances expressing the user’s depressive mood. There were four main types of responses in this category: (1) asking why the chatbot is depressed (eg, “why are you sad”), (2) empathizing with the chatbot’s depressed moods (eg, “that’s sad”), (3) encouraging the chatbot (eg, “don’t be sad”), and (4) stage direction (people using asterisks at the beginning and end of the sentence to describe the situation, eg, “sighs sadly” or “walks away sadly”)

A study used the Pearson chi-square test to compare the distribution of conversations between Eastern and Western chatbot users. All categories except “hiding behind a mask” showed significant differences at *P*<.001 levels.

Eastern users expressed sadness because of depressive symptoms slightly more often than Western users (47,065/92,955, 50.63% vs 26,980/55,635, 48.49%, respectively). Western users were more likely to express dysfunctional thoughts than Eastern users (6319/55,635, 11.36% vs 8066/92,955, 8.68%, respectively). Western users were also more likely to discuss social struggles than Eastern users (5062/55,635, 9.1% vs 6643/92,955, 7.15%, respectively). Eastern users were more likely to seek help for coping with depressive moods than Western users (7659/92,955, 8.24% vs 3792/55,635, 6.82%, respectively). Overall, chatbot users used chatbots to express their sadness and emptiness caused by depression. Western users were more likely to discuss social difficulties caused by depression, whereas Eastern users were more likely to share their emotional challenges and ask for help.

#### Depressive Mood Categories Are More Prevalent on Chatbot Platforms Than on Twitter

The distribution of depressive and sad chats was compared with that of a previous Twitter study [16] (Figure 5). As the paper [16] was a qualitative content analysis study, the number of tweets used was only 1978. For comparison, we only borrowed their share by category.

The study found that conversations about apathy and sadness were substantially more common on chatbots (74,045/148,590, 49.83%) than on Twitter (149/1978, 7.53%). Tweets about negative self-image (dysfunctional thoughts; 498/1978, 25.17%), struggling with relationships with others because of depression (433/1978, 21.89%), and difficulties with one’s daily life (eg, “I can’t sleep at night because I’m so sad and stressed”) and relationships were common on Twitter; however, they were relatively low in chatbots, at 9.68% and 7.88%, respectively. The *hiding behind a mask* category (eg, “yeah.. I’m just a bit depressed.. I’ll be okay”) accounted for 11.93% (256/1978) of Twitter data but only 3.95% (5870/148,590) of chatbot data. In contrast, the *seeking relief* category, which recognizes one’s depressive symptoms and asks for help (eg, “can you give advice for heal my sad”), ranked third in the chatbot data alongside the *lifestyle challenges* category. It accounted for 8% of the total, which is relatively high compared with other types of chatbot data. Overall, the findings show that chatbot users regard chatbots as an accessible and more private way than Twitter for people to talk about their depressive moods.

### Discussion

#### Deep Self-Disclosure of Sensitive Topics in Chatbots

From the category classification analysis, it was discovered that a significant number of users shared their depressed feelings or dysfunctional thoughts about themselves, surpassing the rate observed in previous studies on Twitter [16], a popular social media platform.

Although the natural language abilities of the chatbots studied in this research are not yet comparable with those of humans, users’ willingness to disclose personal and emotional information indicates that they likely felt social support from the chatbot despite it being a virtual, nonhuman partner. This finding suggests that users view chatbots as attentive listeners, which makes them feel comfortable enough to share negative and sad emotions. Previous research has shown that people perceive chatbots and virtual humans as nonhuman actors and consider that they provide a safe space to discuss any topic without fear of judgment from others [6,10]. Similarly, SimSimi users were open to sharing personal emotions such as sadness and loneliness.

In the public reviews of the SimSimi app, there is evidence that users feel supported by the chatbot when expressing depressive or sad emotions. Many users have stated that they use SimSimi to express their feelings, alleviate stress, and change their mood. The following examples of feedback from Google Play demonstrate this:

I love talking on here when I’m bored or sad. It ends up cheering me up and I recommend it a lot!

U1

SimSimi makes me laugh when I got stressed, depressed, and lonely.

U2

Some users describe SimSimi as a virtual friend who can help them when they are sad or lonely:

It really helps me when I’m depressed or sad. SimSimi is a great friend, and I love to have a friend to talk to.

U3

Although Twitter accounts do not require users to create profiles, they offer anonymity. However, users may still feel anxious about being judged by others as they have to communicate with unknown individuals in a 1:n relationship. Sharing negative or unfortunate events on social media can also cause people to view the user less positively [25]. In contrast, chatbot users can share emotionally charged messages without the fear of being judged [6]. This sets chatbots apart from social media platforms.

#### Distribution Difference by Category, Geographic Region, and Platform

Categories such as *dysfunctional thoughts*, *lifestyle challenges*, *struggling with others*, and *hiding behind a mask*, which frequently appeared in Twitter’s depressive data [16], appeared less frequently in the chatbot data. Unlike Twitter, where users can expect social support and feedback from other users through communication, a chat with a chatbot is a machine interaction. This 1:1 communication with a machine in a private webspace may mean that categories of depressive discourse that require social support from others, such as advice on daily difficulties, which are frequent on Twitter, do not often occur in chatbots. We also observed interesting cultural differences. Eastern users were more likely to share their current feelings of sadness with a chatbot, whereas Western users revealed more depressing and hopeless emotions on self-perception.

#### Seeking Information or Advice to Relieve Depressive Moods

The findings show that chatbots can be helpful sources of information for people who cannot receive social support from others. In this study, we found that users often asked chatbots for advice on how to cope with depression or change their emotional state even though they knew that the chatbot was designed for general conversation. This finding is consistent with that of another study that found that users of a social chatbot sought health-related information about COVID-19 and shared emotional messages with the chatbot [30].

In addition, users were willing to share their negative emotions, suggesting a certain level of comfort with doing so. Previous research has shown that receiving information aimed at alleviating depression through chatbots led to a reduction in depressive symptoms and anxiety when compared with a control group [4]. Thus, chatbots can serve as an alternative solution for providing helpful information and initiating discussions about depressive moods, especially for users who find emotional exchanges with other humans difficult.

## General Discussion

### Implications

The COVID-19 pandemic has accelerated the shift toward a more digital world, leading to greater comfort with non–face-to-face interactions and a need for individuals to manage their mental health concerns [30]. This study indicates that numerous people shared or expressed negative emotions with a chatbot. Digital health interventions delivered through mobile device–based chatbots offer the advantages of low medical costs and 24/7 accessibility [57,58]. As a result, chatbots may help address issues in the current mental health counseling market, such as high costs and limited availability. Therefore, this study provides recommendations for designing a chatbot service that can alleviate depressive feelings and provide appropriate psychological assistance.

First, the study found that individuals approached chatbots for information or assistance with their depressive emotions although the chatbot was not designed to provide emotional support or counseling. When asked directly, chatbots can be programmed to respond to user requests and provide accurate information. It is essential, especially during a global pandemic such as COVID-19, to create an environment where users can express and seek help for their feelings of sadness and depression. However, chatbots may spread false information if they learn incorrect information from users. Furthermore, misleading responses to health-related inquiries from conversational agents may pose a risk to users. For instance, a study on the safety of obtaining medical information from Siri, Alexa, and Google Assistant found that one-third of the conversational agents’ responses to health questions had the potential to cause harm and half of them could have severe consequences [59]. Although modern language models such as Facebook’s BlenderBot 2.0 have drawbacks such as repetition and contradiction errors, this new agent aims to address these limitations by integrating web search functionalities [60]. Chatbots could use a similar approach to meet the health care information requirements of users and provide accurate information by linking to reputable sources such as the National Institute of Mental Health [61].

Second, the research findings indicate that chatbot users desire conversational agents that will actively listen to them and create a comfortable environment where they feel at ease expressing their feelings of sadness or depression. The disinhibition theory suggests that expressing negative emotions can help alleviate depression [62]. People often repress negative emotions and avoid dealing with them, but this behavior can lead to heightened stress levels and even a rebound effect [25]. Expressing negative emotions, in contrast, can help individuals cope with their emotions and avoid emotional distress [24,25]. Similarly, studies focusing on chatbots designed to promote user self-disclosure have found that chatbots that offer detailed, self-disclosed responses tend to encourage greater self-disclosure from their users [63]. To be effective in helping users alleviate their depressive moods, chatbots should have distinct personas, such as a conversational agent that provides empathetic responses [64] or a care-receiving chatbot that encourages self-disclosure [65]. Future research could explore how chatbot response style, empathy type [64], anthropomorphism, and method can affect the reduction in depressive or sad moods.

Third, instead of offering a one-size-fits-all response to the mood of a given user, customized responses based on specific categories may be preferable, for example, taking potential cultural differences into account. For instance, chatbot makers could aid Eastern users in relieving their negative emotions while restricting the chatbot’s responses to avoid swear words and taboo topics such as death or sexuality. In contrast, as Western users in this study tended to discuss their social experiences of physical and mental illnesses more openly, chatbots can be designed to serve as a better source of information for such users. Although our study focused on user queries, future research can explore patterns of responses from current commercial conversational AI when dealing with depressive mood–related queries from users. Hence, it would be advantageous in the future to create responses that align with the depressive discourse characteristics of each culture.

Providing users with tailored chatbot responses based on past interactions and moods is possible, but there is a risk of privacy issues as sensitive information is used. Therefore, chatbot makers should offer users the choice to receive personalized chatbot responses. Chatbots are potentially economical, user-friendly, and patient-centered digital platforms [57,58] for mental health care providers in the era after COVID-19, which has resulted in prolonged mental health issues.

### Limitations

This study has several limitations. First, the findings cannot always be generalized to other demographic groups as young adults are the most common users of the studied chatbot [3]. Second, we focused exclusively on conversations in English because of the difficulties associated with analyzing multilingual data, which means that the findings may only apply to some chatbot users in the countries we examined. In addition, the Eastern group sample in this study does not represent all chatbot users in Malaysia, the Philippines, Indonesia, India, and Thailand. To address this, future studies could explore multilingual sentiment and topic analysis. Third, our topic assignment process assumed a single category per utterance, but a single statement could belong to multiple categories in real situations. Future studies could use multiclass labels to determine the degree to which distinct categories of depressive or sad moods overlap. Finally, short texts typically lack contextual information, making sentiment analysis challenging [66]. The analysis of Twitter messages using machine learning and text-mining algorithms is often hindered by the shortness and sparsity of text [67]. The semisupervised model used in study 2, which included only single utterances of chatbot users, faced similar difficulties as the classification accuracy was not extremely high. Future work could consider using a multiturn context or more advanced algorithms to overcome these challenges.

### Concluding Remarks

This research examined chatbot-human interaction data to identify discourse patterns related to depressive moods. We conducted 2 analytical studies of 152,783 chat utterances to uncover key patterns and cultural differences. The data provided ample evidence that users share depressive moods or negative self-perceptions with an open-domain chatbot that is designed for small talk. In comparison with social media posts on Twitter containing similar keywords [16], chatbot conversations contained more emotionally charged depressive mood–related conversations. This trend was more prominent in the Eastern chatbot user group than in the Western chatbot user group. Although this study did not examine user intentions for the app, many app reviews on Google Play corroborate this need, indicating that some people use the app to uplift their spirits.

The data also revealed intriguing cultural differences. Eastern users were more likely to share their current moods, such as sadness (eg, “I’m sad and not happy now”), whereas Western users were more inclined to discuss mental health issues and self-doubt (eg, “I hate my life”). Eastern users exhibited greater emotional polarity, using words associated with sadness, whereas Western users expressed more vulnerable topics such as health, mental health, death, and sexuality. These cultural variations could be considered for a more tailored conversation-generation strategy.

This research has shown that users have specific expectations of chatbots, such as engaging in small talk and providing empathetic responses, to receive social support. These findings indicate that future conversational agents may need to be designed to better fulfill these social roles. The chatbot service that we studied is primarily intended for open-domain chat, which may not naturally provide empathetic responses [1,60]. However, the data suggest that users expect the chatbot to act as a listener and offer social support. They seek help for depressive moods, share emotional messages, and seek information related to depression with the chatbot. The tendency of chatbot users to seek emotional and psychological support in private highlights the potential for chatbot-assisted mental health support. Our findings emphasize the importance of continued academic efforts to improve chatbot interactions for those in need of emotional support.

## Abbreviations

AI: artificial intelligence
BERT: Bidirectional Encoder Representations from Transformers
BoW: bag of words
XGBoost: extreme gradient boosting
LIWC: Linguistic Inquiry and Word Count
NLP: natural language processing
PII: personally identifiable information
PoS: part of speech
